# A Risk Score for Predicting Multiple Sclerosis

**DOI:** 10.1371/journal.pone.0164992

**Published:** 2016-11-01

**Authors:** Ruth Dobson, Sreeram Ramagopalan, Joanne Topping, Paul Smith, Bhavana Solanky, Klaus Schmierer, Declan Chard, Gavin Giovannoni

**Affiliations:** 1 Queen Mary University London; Blizard Institute, Barts and the London School of Medicine and Dentistry, London, United Kingdom; 2 Royal London Hospital, Barts Health NHS Trust, London, United Kingdom; 3 NMR Research Unit, Queen Square Multiple Sclerosis Centre, University College London (UCL) Institute of Neurology, London, United Kingdom; 4 National Institute for Health Research (NIHR) University College London Hospitals (UCLH) Biomedical Research Centre, London, United Kingdom; Heinrich-Heine-Universitat Dusseldorf, GERMANY

## Abstract

**Objective:**

Multiple sclerosis (MS) develops as a result of environmental influences on the genetically susceptible. Siblings of people with MS have an increased risk of both MS and demonstrating asymptomatic changes in keeping with MS. We set out to develop an MS risk score integrating both genetic and environmental risk factors. We used this score to identify siblings at extremes of MS risk and attempted to validate the score using brain MRI.

**Methods:**

78 probands with MS, 121 of their unaffected siblings and 103 healthy controls were studied. Personal history was taken, and serological and genetic analysis using the illumina immunochip was performed. Odds ratios for MS associated with each risk factor were derived from existing literature, and the log values of the odds ratios from each of the risk factors were combined in an additive model to provide an overall score. Scores were initially calculated using log odds ratio from the HLA-DRB1*1501 allele only, secondly using data from all MS-associated SNPs identified in the 2011 GWAS. Subjects with extreme risk scores underwent validation studies. MRI was performed on selected individuals.

**Results:**

There was a significant difference in the both risk scores between people with MS, their unaffected siblings and healthy controls (p<0.0005). Unaffected siblings had a risk score intermediate to people with MS and controls (p<0.0005). The best performing risk score generated an AUC of 0.82 (95%CI 0.75–0.88).

**Interpretations:**

The risk score demonstrates an AUC on the threshold for clinical utility. Our score enables the identification of a high-risk sibling group to inform pre-symptomatic longitudinal studies.

## Introduction

No single factor appears to precipitate the development of multiple sclerosis (MS); a complex interplay of risk factors provides overall risk [1]. Epidemiological data implicates both genetic and environmental factors in disease development. Identifying and studying asymptomatic individuals at high risk of MS provides a powerful opportunity to understand the MS causal cascade. As the MS therapeutic armamentarium increases, the importance of early treatment in influencing long-term outcomes has been realised. Early identification of MS risk provides the opportunity to prevent fixed disability. Development of a predictive tool has the potential to inform longitudinal studies, enriching trial populations with those at high risk of disease.

The majority of people with MS (PwMS) present with a clinically isolated syndrome (CIS), a distinct first episode of demyelination [2]. Radiological abnormalities can be identified in the absence of clinical symptoms, the “radiologically isolated syndrome” (RIS) [3]. Some of those thought to have a RIS show subclinical cognitive impairment similar to the profile of early MS [4], highlighting the prodromal nature of RIS. Approximately two-thirds of those with RIS show new lesions on MRI and one-third develop clinical symptoms of demyelination during mean follow-up times of up to five years [4].

There is a significant genetic contribution to MS. HLA-DRB1*1501 confers a relative risk of MS among heterozygotes of around 3 (6.2 in homozygotes) [5]. A genome-wide association study (GWAS) in 2011 [6] validated 23 non-MHC single nucleotide polymorphisms (SNPs) contributing to MS risk, in addition to identifying a further 29 non-MHC SNPs and 4 MHC alleles [6]. A second GWAS in 2013 [7] increased the number of MS-associated SNPs to 110. Many of the SNPs identified are linked to immune system function.

MS is more common in females than in males, with an estimated relative risk (RR) of 2.62 [8]. A similar pattern is seen in both CIS and RIS [9]. Within regions of temperate climate, MS incidence and prevalence increase with latitude [10]; population-based data demonstrates a correlation between UV exposure and MS prevalence [11, 12]. High serum 25-hydroxyvitamin D (25-OHvD) levels are associated with a lower risk of future MS [13].

Virtually all PwMS have evidence of prior infection with EBV compared to ~94% of age-matched controls [14]. People with high IgG titres against the EBNA-1 EBV epitope have an increased risk of developing MS compared to those with low titres [15, 16]. MS risk is increased in those with a history of symptomatic primary EBV infection (infectious mononucleosis) [17]. Smoking also increases MS risk [18].

Siblings of PwMS have an increased risk of developing MS [19]. This risk is higher in those more closely related to the MS proband; however concordance rates between monozygotic twins of <100% highlight environmental factors in disease aetiology. CSF oligoclonal IgG bands are present in 19% siblings of PwMS compared to 4% healthy controls [20]. Asymptomatic MRI abnormalities suggestive of MS are seen in approximately 10% siblings of PwMS [21, 22] compared to 0.06% of the general population [23]. We suspect that those siblings with a high number of MS risk factors may also have increased probability of CSF oligoclonal bands and/or changes in keeping with MS on magnetic resonance imaging (MRI).

We set out to develop a risk score for the development of MS by integrating genetic and environmental factors known to influence MS risk using techniques described by De Jager et al [24]. We then used this risk score to examine whether siblings occupy an “intermediate” score, between people with MS and healthy controls. Should siblings occupy such an intermediate score, then this would potentially indicate that the risk score provides some indication of overall MS risk. We also aimed to demonstrate that the inclusion of environmental factors into a risk score is able to improve the area under the curve of a score when compared to scores using genetic factors alone, or genetic factors with a single environmental component [24].

Finally we studied whether it is possible to partition the intermediate sibling group into those with a peripheral phenotype in keeping with MS versus healthy controls. We performed a validation step by performing brain MR imaging on a subgroup of participants.

## Materials and Methods

### Sample size calculation

Existing data states that CSF OCBs are seen in 19% siblings of people with MS [20]; it would seem logical that these siblings are at higher risk of MS than those without OCBs. Using this data, 76 siblings are required to give a power of 0.8 and an alpha of 0.05 to detect a difference in CSF OCBs (and hence high MS risk) between unaffected siblings and healthy controls. If 15% siblings have high MS risk, then 111 siblings are required to give power 0.8 and alpha 0.05.

### Subjects

A total of 302 participants were enrolled: 78 probands with MS, 121 of their unaffected siblings (including 6 monozygotic and 3 dizygotic twins), and 103 healthy controls (matched to the sibling group with respect to gender and decade of age) who had no first or second-degree relatives with MS. MS participants were recruited via MS clinics and publicity via local and national MS charities. Siblings were recruited via the proband with MS. Healthy controls were recruited via local recruitment and recruitment drives at various workplaces. This study had ethical approval following review by the regional ethics committee (East London REC 1 (ref. 10/H0704/62). All participants gave informed written consent. Details of participants are given in Table 1.

**Table 1:** Details of participants.

	MS	Unaffected siblings	Healthy controls
**Number**	78	121	103
**Age (mean; SD; range)**	47.26 (11.74; 20–74)	47.24 (12.55; 18–75)	41.22 (11.33; 21–72)^a^
**Gender (M:F; %F)**	8:70 (89.7% F)	38:83 (68.6% F)^b^	33:70 (68.0% F)
**Type of MS (n; %)**	• RRMS: 45 (69.2%) • SPMS: 16 (20.5%) • PPMS: 7 (9.0%)		
**Treatment (n; %)**	37 (47.4%)^c^		
**EDSS (mean; range)**	3.79 (0–8.5)		

a: Healthy controls were significantly younger than people with MS (p<0.0005) and their siblings (p = 0.003), one way ANOVA.

b: Probands with MS were significantly more likely to be female than their unaffected sibling (p = 0.0005) and healthy controls (p = 0.0006), Fisher’s exact test. There was no significant difference in the gender distribution of the groups between siblings and healthy controls.

c: 3 patients on Avonex, 2 on betaferon, 14 on Rebif, 14 on copaxone, 4 on Natalizumab and 2 on mitoxantrone (last dose >4 months ago for both).

All participants were seen in person by a single investigator (RD). All patients and unaffected siblings underwent neurological examination and a structured interview. Details regarding place and month of birth, self-reported history of infectious mononucleosis, smoking history, co-morbid medical conditions, family history and medication (including vitamin supplementation) were recorded. Blood draw and urine sample collection were performed on the same day.

### Laboratory analysis

All serum samples were divided into aliquots and frozen at -80°c within 2 hours of blood draw. Peripheral blood mononuclear cells were separated and stored in liquid nitrogen on the day of blood draw. Analysis was performed in bulk on the day of thawing. Samples were not subject to repeated freeze thaw cycles.

Anti-EBNA-1 IgG titres were measured using a commercially available ELISA (DiaSorin; Salugia, Italy). Current smoking status was assessed using a commercially available cotinine ELISA (Calbiotech; California, USA), a concentration of 3.08ng/ml was used to define current smokers [25]. Serum 25-OHvD levels were measured using liquid chromatography-tandem mass spectrometry (Department of Clinical Biochemistry, Royal London Hospital, Whitechapel). Serum 25-OHvD was deseasonalised to the day of sampling according to standard methods [26].

DNA was isolated from whole blood prior to genotyping using the Illumina immunochip [27]. The immunochip provides SNP data for all SNPs associated with MS and a number of other autoimmune diseases in GWAS; SNP data for the MS-associated alleles was isolated for each individual and analysed.

### Construction of a risk score

Odds ratios for MS associated with each risk factor were derived from the existing literature (Table 2). Where available in the published literature, meta-analyses were used. Where meta-analyses were not available, the odds ratios were taken from the largest available studies. Anti-EBNA-1 IgG titres were assigned to quintiles derived using the combined healthy control and sibling titres as the reference group [16]. As there are no published estimates for the odds ratio of MS for each epoch of age, and there was no clear threshold age beyond which MS risk declines rapidly, the decision was taken not to include age as a risk factor in this MS risk score.

**Table 2:** Summary of relative risks used in the calculation of an overall risk score. The risk associated with carriage of the HLA-DRB1*1501 haplotype is given; for the risks associated with other HLA haplotypes and non-MHC SNPs see appendix 1.

Risk factor	Relative risk used in risk score calculation	Log value used in additive model
**Gender (9)**	Female: 2.22	0.35
**Month of birth (11)**	• April: 1.08 • May: 1.09 • October: 0.95 • November: 0.90	• 0.03 • 0.04 • -0.02 • -0.05
**Previous infectious mononucleosis (17)**	2.17	0.34
**Quintile of IgG against EBNA-1 (16)**	• Undetectable titres: 0.33 • Q1 (lowest; reference): 1.0 • Q2: 2.6 (0.7–9.2) • Q3: 3.2 (1.0–10.4) • Q4: 5.1 (1.5–17.6) • Q5: 9.4 (2.5–35.4)	• -0.48 • 0.00 • 0.00 • 0.51 • 0.71 • 0.97
**Quintile of serum 25-OHvD (13)**	• Q1 (lowest; <63.2nmol/l): 1.0 (reference) • Q2 (63.3–75.3nmol/l): 0.57 (0.3–1.07) • Q3 (75.4–84.8nmol/l): 0.57 (0.3–1.07) • Q4 (84.9–99.1nmol/l): 0.74 (0.4–1.36) • Q5 (>99.2nmol/l): 0.38 (0.19–0.75)	• 0.00 • 0.00 • 0.00 • 0.00 • -0.42
**Smoking status (18)**	Ever smoking: 1.52	0.18
HLA-DRB1*1501 haplotype (6)	• Heterozygote: 3.1 • Homozygote: 6.2	• 0.49 • 0.79

Deseasonalised serum 25-OHvD levels were assigned to quintiles according to the values specified by Munger et al [13]. The only quintile that carried an odds ratio of MS significant different to that of the lowest quintile was the highest quintile (Q5); for full details of all odds ratios see Table 2. Participants who either described a smoking history and/or those with a cotinine level greater than 3.08ng/ml were coded as “ever smoking”. 16 participants (3 MS, 5 siblings and 8 HC) had a positive cotinine ELISA despite reporting no current smoking. Only 1 participant described a current smoking habit but had a negative cotinine ELISA. All participants bar one reported a place of birth in the UK; therefore latitude of birth was not included as a factor in the overall risk score.

The log values of the odds ratios from each of the risk factors were combined in an additive model [24] to provide an overall score. The weighting given to each of the non-genetic risk factors and possession of the HLA-DRB1*1501 allele is given in Table 2. Details of the weighting given to all genetic risk factors is provided in Table 3. Where a risk factor was not present, a value of 0 was assigned for this risk factor. The total sum of all log odds ratio values was the risk factor score for an individual.

**Table 3:** SNPs associated with MS available on the Illumina Immunochip.

SNP identified in GWAS	Associated candidate gene	Risk allele	Odds Ratio associated with risk allele (6)
**rs1315388**	HLA-DRB1*1501	A	3.10
**rs4648356**	MMEL1	C	1.14
**rs11810217**	EV15	A	1.15
**rs11581062**	VCAM1	G	1.12
**rs1335532**	CD58	A	1.22
**rs1323292**	RGS1	A	1.12
**rs7522462**	C1orf106(KIF21B)	G	1.11
**rs12466022**	no gene	C	1.11
**rs7595037**	PLEK	A	1.11
**rs17174870**	MERTK	G	1.11
**rs10201872**	SP140	A	1.14
**rs11129295**	EOMES	A	1.11
**rs669607**	no gene	C	1.13
**rs2028597**	CBLB	G	1.13
**rs2293370**	TMEM39A/CD80	G	1.13
**rs9282641**	CD86	G	1.21
**rs2243123**	IL12A	G	1.08
**rs228614**	NFKB1	G	1.09
**rs6897932**	IL7R	G	1.11
**rs4613763**	PTGER4	G	1.20
**rs2546890**	IL12B	A	1.11
**rs12212193**	BACH2	G	1.09
**rs802734**	THEMIS	A	1.10
**rs11154801**	MYB/AHI1	A	1.13
**rs17066096**	IL22RA2	G	1.14
**rs13192841**	no gene	A	1.10
**rs1738074**	TAGAP	G	1.13
**rs354033**	ZNF746	G	1.11
**rs1520333**	IL7	G	1.10
**rs4410871**	MYC	G	1.11
**rs2019960**	PVT1	G	1.12
**rs3118470**	IL2RA	G	1.12
**rs1250550**	ZMIZ1	A	1.10
**rs7923837**	HHEX	G	1.10
**rs650258**	CD6	G	1.12
rs630923*	CXCR5	C	1.12
**rs1800693**	TNFRSF1A	G	1.12
**rs10466829**	CLEC1	A	1.09
**rs12368653**	CYP27B1	A	1.10
**rs949143**	ARL6IP4	G	1.08
**rs4902647**	ZFP36L1	G	1.11
**rs2300603**	BATF	A	1.11
**rs2119704**	GALC/GPR65	C	1.22
**rs2744148**	SOX8	G	1.12
**rs7200786**	CLEC16A	A	1.15
**rs13333054**	IRF8	A	1.11
**rs9891119**	STAT3	C	1.11
**rs180515**	RPS6KB1	G	1.09
**rs7238078**	MALT1	A	1.12
**rs1077667**	TNFRSF14	G	1.16
**rs8112449**	TYK2/CDC37	G	1.08
**rs874628**	MPV17L2	A	1.11
**rs2303759**	DKKL1	C	1.11
**rs2425752**	CD40	A	1.11
**rs2248359**	CYP24A1	G	1.12
**rs6062314**	ZBTB46/TNFRSF6B	A	1.16
**rs2283792**	MAPK1	C	1.10
rs140522*	SCO2	A	1.10

Odds ratios taken from (6).

Two models were derived, the initial model used the genetic data (i.e. log odds ratio) from the HLA-DRB1*1501 allele only. Following this, a second model was derived using data from all MS-associated SNPs identified in the 2011 GWAS [6]. The decision was taken not to use all SNPs identified in the 2013 GWAS [7], as the magnitude of the genetic score would far outweigh any environmental contribution to the score if all 110 genetic variants were included, given the simple additive model. The risk score derived using all genetic information was used to select participants for MRI.

### Validation of the risk score

#### Magnetic resonance imaging

To determine if the MS risk score in siblings correlated with the appearance of asymptomatic pathology, T2-weighted MR brain imaging was acquired to look for MS-like white matter lesions and single voxel proton spectroscopy undertaken to look for metabolic changes found in people with MS. 10 siblings with a high risk score and 10 siblings with a low risk score underwent imaging studies. Scanning was performed on a single Philips Achieva 3 Tesla MRI system with the manufacturer’s product 32-channel head coil. All scans were acquired within a three month period, during which there were no major hardware upgrades or replacements. All subjects underwent scanning using the same protocol. PD/T2-weighted images were acquired using a 2D turbo spin echo (2D-TSE) sequence (field of view 240 x 180mm, voxel size 1 x 1x 3mm over 50 slices; echo train length (ETL) = 10, repetition time (TR) = 3500ms, echo time (TE) = 19/85ms, number of excitations (NEX) = 1; total scan time = 4 minutes). Image interpretation was performed by assessors blind to the risk status of participants. Images were assessed by a radiologist (PS) and a neurologist trained in image interpretation (KS) for the presence of T2 hyperintensities in keeping with demyelination using internationally accepted criteria developed by MAGNIMS [28,29]. Single voxel proton spectroscopy (TE = 32ms, TR = 2s, 20x10x10 mm voxel, PRESS localisation with MOIST water suppression and an identical water reference scan with no MOIST water suppression applied from the same voxel) was undertaken in normal appearing brain white matter. LCModel was used to analyse the spectra and to estimate the concentration of creatine and phosphocreatine, glycerophosphocholine and phosphochocholine, and N-acetyl-aspartate and N-acetyl-aspartyl-glutamate, and myo-inositol, using the water reference scan for quantification.

#### Statistical analysis

Statistical analysis was performed using PASW Statistics 18 (SPSS; IBM UK). Normality was assessed using a Shapiro-Wilk test. Scores between three groups were compared using a one-way ANOVA prior to post-hoc testing using a Bonferroni correction. Receiver operating characteristic (ROC) curves were generated using standard techniques.

## Results

### Risk score

**Fig 1:**
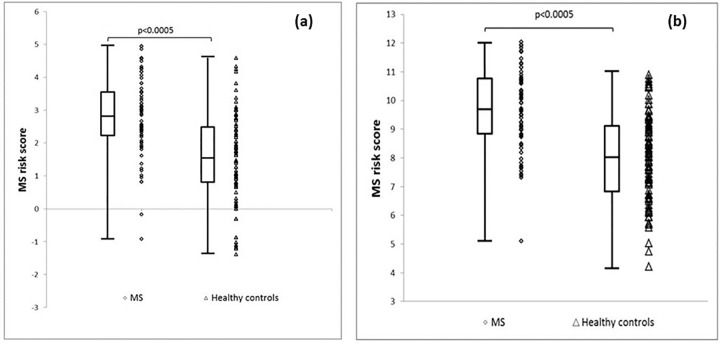
Combined scatter and box-and-whisker plot demonstrating the MS risk score distribution between people with MS and healthy controls. The box indicates the interquartile range, bisected by the median, and the whiskers the range. **(a)** Where HLA-DRB1*1501 is the only genetic information used to derive the risk score. **(b)** Where full genetic information is used to derive the MS risk score.

**Fig 2:**
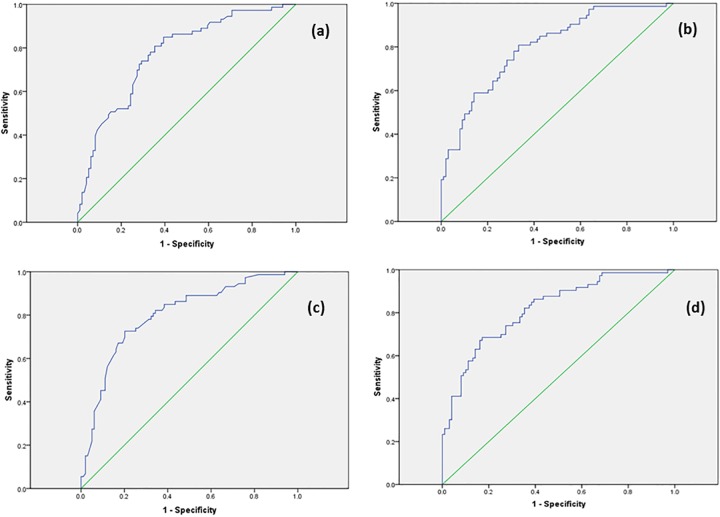
A receiver operating characteristic (ROC) curve generated by the MS risk score when the score of people with MS is compared to that of healthy controls. **(a)** Including HLA-DRB1*1501 only. **(b)** Including full genetic information. **(c)** Including genetic information from HLA-DRB1*1501 only, and excluding serum 25-hydroxyvitamin D levels. **(d)** Including full genetic information, and excluding serum 25-hydroxyvitamin D levels.

The risk score was initially generated using the genetic information regarding HLA-DRB1*1501 only in addition to all of the environmental factors. The distribution of the MS risk score was Gaussian for all groups. Details regarding the risk scores are given in Table 4. There was a significant difference in the risk score between people with MS and healthy controls (p<0.0005) (Table 4 and Fig 1A). A ROC curve comparing people with MS with healthy controls generated an area under the curve (AUC) of 0.77 (95% CI 0.70–0.84) (Table 4 and Fig 2A).

**Table 4:** Risk scores and area under a ROC curve for each group and risk score respectively.

	MS (n = 73)	Siblings (n = 107)	Healthy controls (n = 99)	Area under curve (95% CI)
Risk score including genetic contribution from HLA-DRB15*1501 only (mean; SD)	2.82 (1.18)^a^	1.98 (1.38)^b^	1.53 (1.34)	0.77 (0.70–0.84)
**Risk score including genetic contribution from all MS risk alleles (mean; SD)**	9.71 (1.38)^c^	8.83 (1.47)	8.00 (1.49)	0.80 (0.74–0.87)
Risk score including genetic contribution from HLA-DRB1*1501 only; excluding serum 25-OHvD level (mean; SD)	3.13 (1.12)	2.16 (1.33)	1.74 (1.32)	0.80 (0.73–0.87)
**Risk score including genetic contribution from all MS risk alleles; excluding serum 25-OHvD level (mean; SD)**	10.02 (1.38)	9.00 (1.44)	8.20 (1.48)	0.82 (0.75–0.88)

a: p<0.0005 for difference between MS and siblings and MS and HC

b: p = 0.042 for difference between siblings and HC

c: p<0.0005 for difference between MS and siblings, MS and HC and siblings and HC.

The contribution from all MS risk alleles was then used to derive the MS risk score (Table 3). There was a significant difference in the risk score between people with MS and healthy controls (p<0.0005) (Table 3 and Fig 1B). A ROC curve comparing people with MS with healthy controls generated an improved AUC of 0.80 (95%CI 0.74–0.87) (Table 4 and Fig 2B).

People with MS had significantly higher deseasonalised serum 25-OHvD than unaffected siblings and healthy controls; the patients with MS were taking vitamin D supplementation at higher rates than either their siblings or healthy controls (data not shown). The risk scores were therefore calculated without the contribution from 25-OHvD. Under these conditions, when the genetic contribution from HLA-DRB1*1501 only was included, the AUC was 0.80 (95% CI 0.73–0.87), and when all genetic information was included the AUC increased to 0.82 (95% CI 0.75–0.88) (Table 4 and Fig 2C and 2D).

### Risk score: siblings

**Fig 3:**
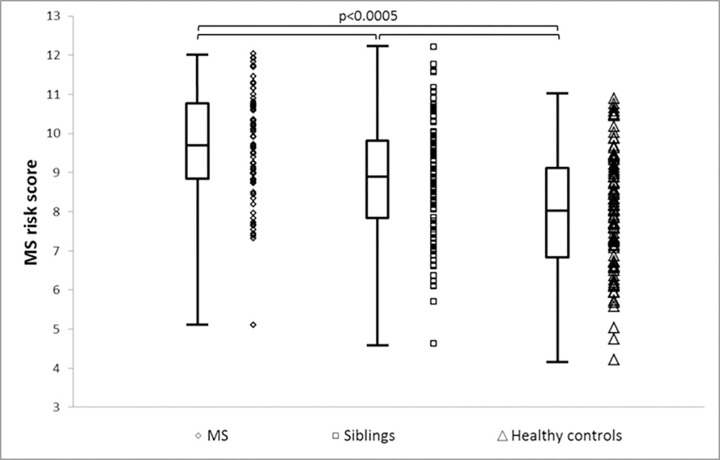
Combined scatter and box-and-whisker plot demonstrating the MS risk score distribution between people with MS and healthy controls. The box indicates the interquartile range, bisected by the median, and the whiskers the range. Full genetic information is used to derive the MS risk score.

The sibling group appeared to occupy an intermediate risk score. There was a significant difference in the risk scores using HLA type between the three groups (p<0.0005; one-way ANOVA) (Table 4 and Fig 3). People with MS had a significantly higher MS risk score than their unaffected siblings (p<0.0005), and unaffected siblings had higher scores than healthy controls (p = 0.042) (Table 4 and Fig 3). The same was true when all MS risk alleles were used to derive the risk score, with post-hoc testing demonstrated significant differences between all pairwise combinations (p<0.0005 for all comparisons).

#### Using the risk score to identify high risk siblings

The risk score was partitioned into 7 categories based on the risk score distribution within that group (+/- 0.25, 0.75 and 1.25 SD from the mean). Comparisons were carried out between the sibling group and the healthy control group. As would be expected, when the lowest septiles of the sibling group were selected, the risk score was significantly different from that of healthy controls. When septiles 1–5 of the sibling group were selected then the groups did not significantly differ (sibs mean score 1.33 vs HC 1.51). When septiles 1–6 of the sibling group were selected, again the groups did not significantly differ (sibs mean score 1.68 vs HC 1.51). When the full sibling group was included the groups significantly differed (see above), indicating that it was only the highest septile of the sibling group that gave rise to the overall difference in mean values.

#### Using these data to inform future longitudinal studies

Given that the group consisting of septiles 1–6 of the sibling group have a risk score distribution not significantly different to that of healthy controls, it would seem reasonable to use siblings with risk scores in the highest septile to inform power calculations for enriching pre-symptomatic trials. From our data this would equate to 10.3% siblings.

#### MRI

One participant in the group with high MS risk score developed clinically definite MS prior to the imaging stage; this participant did not have exploratory imaging. There was no significant difference between the high and low risk imaging groups in terms of number or location of lesions. One participant in each group had changes that could be described as “possible demyelination” according to accepted criteria [28,29]. Single voxel spectroscopy did not demonstrate any difference in the concentration of either NAA, NAA-G or myo-inositol in normal appearing white matter between siblings with high and low risk scores.

## Discussion

Developing a tool to stratify MS risk may enable preventative trials to be sufficiently powered, something that is currently not possible. The MS risk score reported here demonstrates performance characteristics at the threshold accepted for clinical utility. ROC analysis demonstrated an area under the curve score of 0.82, i.e. just below 0.85 [30], the figure generally accepted as being clinically useful. Our study demonstrates that it is possible to create a multivariate MS risk score with real potential to inform the clinical discussion with relatives of people with MS beyond currently available tools.

Our finding that 90% of siblings lie in septiles 1–6 with overall risk score distribution not significantly different from matched healthy controls gives an indication as to how these data can be used to inform longitudinal clinical trials. Enriching such trials with siblings at the highest risk of MS at baseline would potentially reduce the number of participants that require follow up. By screening participants using an MS risk score at baseline, only the 10% of siblings in the highest risk groups need by subject to intensive monitoring. Refining intensive monitoring to this 10% has clearly significant implication for feasibility of longitudinal studies.

An alternative means of identifying people at high risk of developing MS could be to screen large numbers of potential participants using MRI to identify those with radiologically isolated syndrome, RIS. However, MRI screening of large numbers of people is both expensive and time consuming for both participants and investigators, whereas many of the factors used in our risk score can be determined from clinical history, with the rest amenable to batch analysis at relatively low cost. In addition, the disease process may well be already established in those with RIS [1]. Our risk score therefore provides a feasible method to detect those with truly asymptomatic disease in large population-based studies.

Whilst the validation step using MRI markers of MS risk did not demonstrate any significant differences between the siblings with high risk score and those with low risk score, longitudinal studies with years of follow up to determine both MRI and clinical conversion rates are the only accurate way to determine the accuracy of any predictive score. Our study provides evidence that it is feasible to develop such a score and hints that it may be worthy of further study.

The risk score can also contribute to our understanding of the causal cascade that eventually results in MS onset. The AUC improves as increasing numbers of SNPs MS are included; however it seems unlikely that expanding the genetic contribution will eventually result in a “perfect” score. It seems likely that consideration of gene-environment interactions will result in meaningful improvement in such a score.

Studies to examine gene-environment interactions would have to be multinational collaborations over many decades, thereby posing significant problems of feasibility. An illustrative model implied a marked potential effect of such interactions on the OR calculated by GWAS, with an increase in the OR of up to 16.8 from a GWAS estimated OR of 1.3 [31]. Other potentially important contributors to MS risk are rare alleles associated with large effect sizes. These low frequency variants are not present on current SNP genotyping arrays. Individual disease risk may be influenced by rare or private (confined to one individual/family) mutations.

One environmental factor that has been shown to be associated with later MS risk is childhood obesity [32]; childhood weight also interacts with a history of infectious mononucleosis [33]. However, we did not have access to the medical records of the majority of participants and so had no accurate measure of BMI in childhood. There is good evidence that on an individual basis there is poor recollection of childhood weight [34], and that recollection may be affected by a number of factors including cognitive ability, which could differ significantly between groups and not be possible to control for in this study. We therefore decided not to include this in our risk score; however it is likely that the risk score could be improved if an accurate measure of childhood weight was available for future participants.

Whilst the decision not to include age as an MS risk factor was valid in this pilot study, age is an important factor in any longitudinal study. MS risk declines with increasing age after a threshold age; people aged >60 years are unlikely to develop clinically definite MS within their lifetime and so have low risk due to age alone. We would suggest that if this risk score were to be used to enrich prevention trials, such trials should exclusively enrol younger participants under a threshold age where the risk of developing MS remains relatively high. In this exploratory study, we suspect that the relatively high mean age of siblings reduced the risk of asymptomatic demyelinating lesions in this group, thus contributing to the non-significant MRI data.

Whilst major known genetic and environmental risk factors have been included in the model described here, it appears evident significant further risk factors have yet to be identified. It also raises questions about risk factors for developing an MS-like pathological process and risk factors for this manifesting as neurological deficits, and these need not be identical.

In the sub-groups who underwent MRI, no differences in T2-weighted lesion loads or brain white matter metabolite concentrations was observed. With due caution given the relatively small number of participants who were imaged, and it may be that imaging the entire cohort is needed to fully assess the distribution of risk, this observation suggests that even in those at relatively high risk of developing MS, further initiating event(s) or acquired risk factor(s) are required before a fully fledged neuroinflammatory process is initiated that is detectable by MRI.

The current model needs to be validated in a larger cohort, but it is also likely that further factors–and their interaction(s)–will have to be discovered to create a more definitive risk score. Clearly longitudinal follow-up of selected groups, stratified according to estimated risk is the gold standard to validate any risk score, however such studies are require proof-of-concept preliminary studies, such as this one, to enable power calculations to be performed. However, as it stands our current model appears to be powerful enough to test hypotheses regarding MS development and has potential to enable directed preventative studies.
